# Granulomatosis With Polyangiitis Misdiagnosed as Tuberculosis: A Case Report

**DOI:** 10.7759/cureus.71834

**Published:** 2024-10-19

**Authors:** José Diogo Martins, Catarina Soares, Cátia Barreiros, Filipa Teixeira, Manuel Ferreira

**Affiliations:** 1 Internal Medicine, Unidade Local de Saúde do Alto Minho, Viana do Castelo, PRT; 2 Rheumatology, Unidade Local de Saúde do Alto Minho, Viana do Castelo, PRT; 3 Internal Medicine, Unidade Local de Saude do Alto Minho, Viana do Castelo, PRT

**Keywords:** anca-associated vasculitis, granulomatosis with polyangiitis, necrotizing granulomas, pulmonary cavitation, rituximab

## Abstract

Granulomatosis with polyangiitis (GPA), formerly known as Wegener’s granulomatosis, is a rare autoimmune disorder characterized by necrotizing vasculitis affecting small to medium-sized vessels. This condition most commonly affects the lungs, kidneys, and upper respiratory tract. Early recognition and treatment are critical to prevent severe complications and improve prognosis. This report presents the case of a 48-year-old male with GPA, with primary pulmonary and articular involvement, who presented with respiratory symptoms and was initially misdiagnosed as having pulmonary tuberculosis due to cavitary lesions on imaging.

## Introduction

Granulomatosis with polyangiitis (GPA), previously known as Wegener’s granulomatosis, is a rare and systemic necrotizing vasculitis predominantly affecting small to medium-sized vessels [1,2]. The condition is part of the spectrum of antineutrophil cytoplasmic antibody (ANCA)-associated vasculitides, which also includes microscopic polyangiitis and eosinophilic granulomatosis with polyangiitis (EGPA) [3]. GPA is characterized by granulomatous inflammation and necrotizing vasculitis, predominantly targeting the respiratory tract and kidneys. While its etiology remains poorly understood, it is thought to involve both genetic predisposition and environmental and immunological factors that culminate in an abnormal autoimmune response [3].

GPA commonly affects the upper and lower respiratory tracts, leading to chronic sinusitis, nasal septal perforation, and pulmonary involvement, including nodules, cavitary lesions, and alveolar hemorrhage. The disease can also affect other organs as well, particularly the kidneys, where it may present as rapidly progressive glomerulonephritis. Untreated GPA can lead to irreversible organ damage and has historically been associated with high morbidity and mortality. Advances in immunosuppressive therapies, particularly the introduction of rituximab, have markedly improved the prognosis of patients with GPA [4].

The diagnosis of GPA is based on clinical presentation, imaging, and laboratory findings, including the detection of ANCA, specifically anti-proteinase 3 (PR3) antibodies, which are present in 80-90% of GPA patients. However, GPA can be challenging to diagnose, as its clinical features overlap with other conditions, particularly infectious diseases such as tuberculosis, which may present with similar pulmonary manifestations, including cavitary lung lesions. Given the potential for misdiagnosis, early recognition and treatment of GPA are critical to prevent disease progression and complications. Delayed or inadequate treatment may result in life-threatening conditions such as pulmonary hemorrhage, renal failure, and disseminated vasculitis. The complexity of GPA’s presentation, coupled with its association with serious morbidity, underscores the need for heightened awareness and multidisciplinary management, particularly when encountering atypical presentations [1,5].

This report presents a case of GPA with prominent pulmonary involvement initially misdiagnosed as tuberculosis. It highlights the diagnostic challenges posed by GPA and emphasizes the importance of a thorough differential diagnosis in patients presenting with cavitary lung lesions.

## Case presentation

A 48-year-old male construction worker presented to the Diagnostic Center for Pneumology with a two-month history of productive cough, weight loss (~6 kg), night sweats, and generalized joint pain. A chest X-ray performed at that time revealed a cavitary lesion in the right upper lobe. Due to a clinical suspicion of tuberculosis, several sputum samples were obtained for bacilloscopy and microbiological testing in February and March; however, no pathogens were identified. Despite the absence of microbiological confirmation, empirical anti-tuberculosis treatment with HRZE (H = isoniazid, R = rifampin, Z = pyrazinamide, E = ethambutol) was initiated based on the high clinical suspicion.

**Figure 1:**
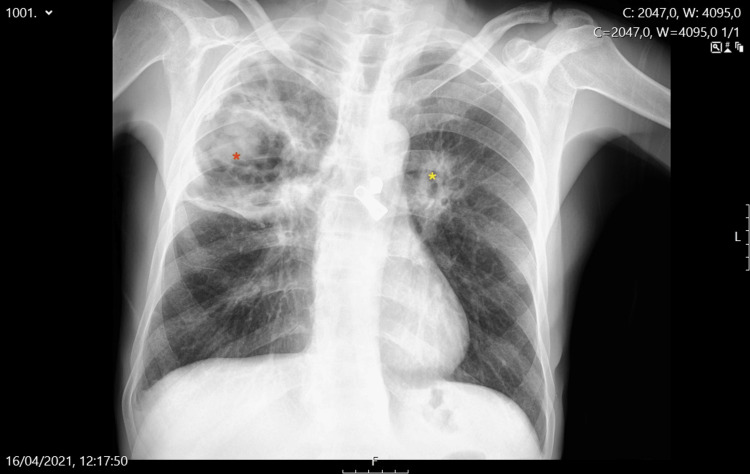
Chest radiograph at hospital admission showing cavitary lesions The chest radiograph taken on April 16, 2021, demonstrated a large cavitary lesion in the right upper lobe (red asterisk) and a smaller cavitary lesion in the left lung (yellow asterisk). These findings were later associated with the diagnosis of granulomatosis with polyangiitis (GPA)

The patient's condition deteriorated, with increasing fatigue and anorexia, leading to his admission to the hospital. A chest X-ray showed multiple cavitary lesions, particularly in the right upper lobe (Figure 1). Laboratory tests revealed normocytic anemia (hemoglobin 9.0 g/dL), elevated C-reactive protein (20.41 mg/dL), and mild hyponatremia (serum sodium 132 mmol/L). A CT scan confirmed cavitary lesions with air-fluid levels, further raising suspicion of tuberculosis.

**Figure 2:**
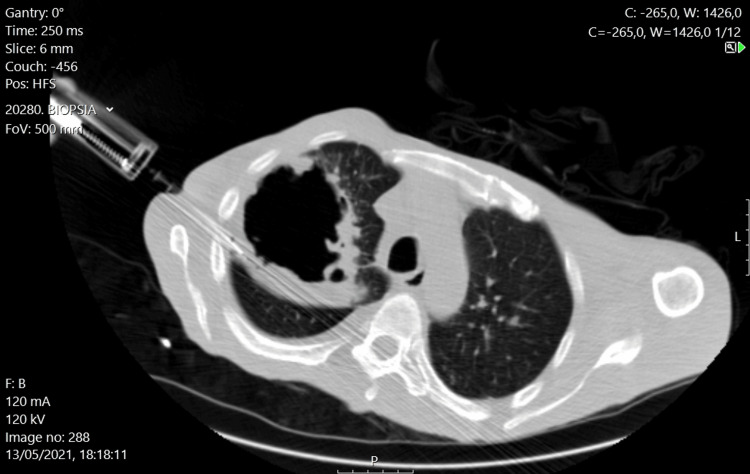
CT-guided percutaneous lung biopsy in the patient Axial CT image showing a percutaneous biopsy needle inserted into a cavitary lesion wall in the right upper lobe of the lung. The biopsy was performed to investigate the nature of the lesion, and histology revealed a granulomatous inflammatory process CT: computed tomography

During hospitalization, the patient was maintained on anti-tuberculosis treatment. The blood cultures were negative either for bacterial or mycobacterial infection. A bronchoalveolar lavage (BAL) was performed, which returned negative for mycobacteria, as well as for bacterial and fungal infections. A biopsy of the cavitary lesion wall was also performed, which indicated a granulomatous inflammatory process (Figure 2). The patient remained clinically and analytically unresponsive to anti-tuberculosis therapy, prompting further investigations. Serology for antineutrophil cytoplasmic antibodies (ANCA) was positive, with elevated anti-PR3 titers (113 U/mL; reference <3 U/mL), hinting at an alternative diagnosis of GPA.

After a multidisciplinary meeting with Rheumatology, Infectious Disease, and Radiology, the diagnosis of GPA was entertained and the HRZE regimen was discontinued. Before initiating immunosuppressive therapy, infectious screening was conducted, including tests for cytomegalovirus (CMV), hepatitis B and C, HIV, and interferon-gamma release assay (IGRA) for tuberculosis (T-SPOT.TB), all of which returned negative. Given the negative screening results, corticosteroid therapy (1 mg/kg of prednisolone) and rituximab were initiated [6].

**Figure 3:**
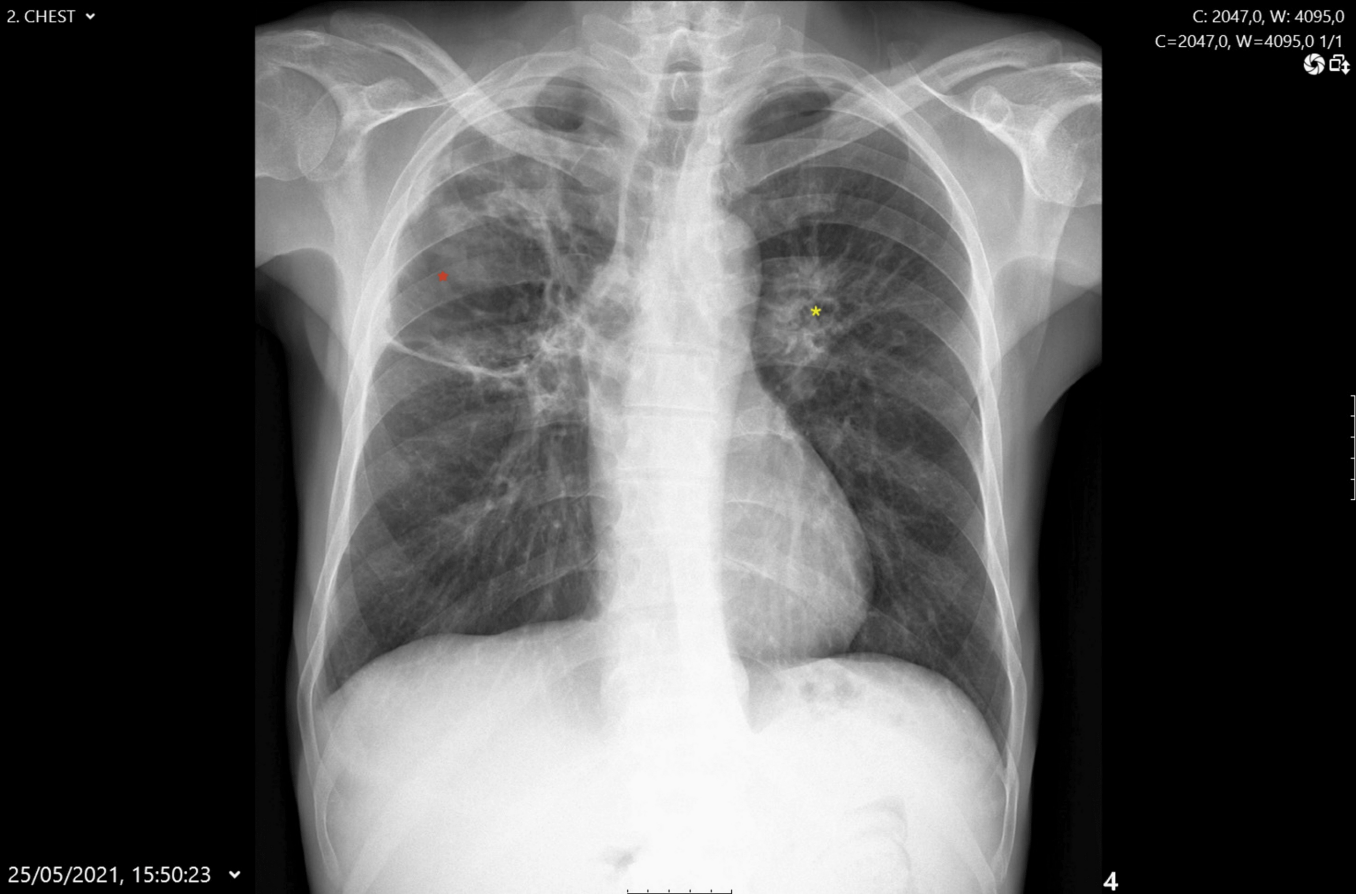
Chest radiograph following initiation of immunosuppressive therapy Post-immunosuppressive therapy chest radiographs taken on May 25, 2021, in anteroposterior view. The images show a reduction in the thickness of the cavitary wall, particularly in the right upper lobe (red asterisk) and left lung (yellow asterisk), with the cavitations still present. These findings suggest a reduction in the inflammatory activity following treatment with corticosteroids and rituximab, consistent with granulomatosis with polyangiitis (GPA)

The patient showed clinical improvement with a reduction in respiratory symptoms, including cough and night sweats. There was also a significant reduction in inflammatory markers thereafter. A follow-up radiograph (Figure 3) showed a reduction in the thickness of the cavitary walls, which was interpreted as a positive indicator of inflammatory activity reduction, although the cavitary lesions persisted.

The patient remained hospitalized for a total of six weeks, during which he experienced progressive clinical improvement, and was discharged in stable condition. Two months after discharge, the patient developed a complication of pneumonia and was readmitted to the hospital. However, instead of following the proposed plan, the patient signed out against medical advice. Tragically, he was found dead at his home shortly after being discharged.

## Discussion

This case highlights the diagnostic challenges associated with GPA, particularly in patients presenting with cavitary pulmonary lesions. In this instance, the patient's imaging findings were highly suggestive of tuberculosis, leading to the initiation of empirical anti-tuberculosis therapy despite negative microbiological results. The diagnosis of GPA that was eventually reached underscores the importance of considering vasculitis in the differential diagnosis of cavitated lung lesions, especially in patients with systemic symptoms and those who fail to respond to conventional treatments for suspected infections.

ANCA testing plays a pivotal role in the diagnosis of GPA, as elevated anti-PR3 titers are a key marker of this disease. The prompt initiation of corticosteroids and rituximab led to clinical improvement, although the persistence of cavitary lesions indicates that long-term management and close follow-up are necessary to monitor disease progression and prevent further complications [6,7].

## Conclusions

This report underscores the importance of considering GPA in patients presenting with cavitary lung lesions that mimic tuberculosis, especially in the absence of microbiological confirmation. Early recognition and initiation of appropriate immunosuppressive therapy are key in preventing the progression of GPA and reducing morbidity. The unfortunate outcome in our patient despite his initial response to treatment highlights the need for careful monitoring of patients with GPA and the potential for severe complications even after discharge.
